# Supplementary material for: Hyper Cross-Linked Polymers as Additives for Preventing Aging of PIM-1 Membranes
Source: Membranes (Basel). 2021 Jun 23;11(7):463. doi: 10.3390/membranes11070463 (PMC8305886; doi:10.3390/membranes11070463)
Supplement: Supplementary file 1 [file membranes-11-00463-s001.zip › membranes-1249577-SI.pdf]

membranes

Supplementary Materials

Hyper Cross-Linked Polymers as Additives for Preventing Aging of PIM-1 Membranes

Federico Begni[1], Elsa Lasseuguette[2], Geo Paul[1], Chiara Bisio[1,3], Leonardo Marchese[1], Giorgio Gatti[1,*] and Maria-Chiara Ferrari[2,*]

Table S1. Elemental analysis performed via EDX spectroscopy on ABT01 and ABT02 materials. Five measurements were performed on each sample and the mean values are here reported.

Element	ABT01 (wt %)	ABT02 (wt %)
**C**	98.50	98.47
**Al**	0.75	0.64
**Br**	0.63	0.88
